# *In Vitro* Antibacterial Activity of Teixobactin Derivatives on Clinically Relevant Bacterial Isolates

**DOI:** 10.3389/fmicb.2018.01535

**Published:** 2018-07-11

**Authors:** Estelle J. Ramchuran, Anou M. Somboro, Shimaa A. H. Abdel Monaim, Daniel G. Amoako, Raveen Parboosing, Hezekiel M. Kumalo, Nikhil Agrawal, Fernando Albericio, Beatriz G. de La Torre, Linda A. Bester

**Affiliations:** ^1^Biomedical Resource Unit, School of Laboratory Medicine and Medical Sciences, College of Health Sciences, University of KwaZulu-Natal, Durban, South Africa; ^2^Peptide Research Group, School of Chemistry and Physics, University of KwaZulu-Natal, Durban, South Africa; ^3^Department of Virology, National Health Laboratory Service, University of KwaZulu-Natal, Durban, South Africa; ^4^Discipline of Medical Biochemistry, School of Laboratory Medicine and Medical Science, University of KwaZulu-Natal, Durban, South Africa; ^5^KRISP, College of Health Sciences, University of KwaZulu-Natal, Durban, South Africa; ^6^CIBER-BBN, Networking Centre on Bioengineering, Biomaterials and Nanomedicine, and Department of Organic Chemistry, University of Barcelona, Barcelona, Spain

**Keywords:** teixobactin derivatives, biological activity, antimicrobial agents, resistant bacteria, antimicrobial peptides, *in silico* analysis

## Abstract

Methicillin-resistant *Staphylococcus aureus* (MRSA) and vancomycin-resistant enterococcus (VRE) are included on the WHO high priority list of pathogens that require urgent intervention. Hence emphasis needs to be placed on developing novel class of molecules to tackle these pathogens. Teixobactin is a new class of antibiotic that has demonstrated antimicrobial activity against common bacteria. Here we examined the antimicrobial properties of three Teixobactin derivatives against clinically relevant bacterial isolates taken from South African patients. The minimum inhibitory concentration (MIC), the minimal bactericidal concentration (MBC), the effect of serum on MICs and the time-kill kinetics studies of our synthesized Teixobactin derivatives (3, 4, and 5) were ascertained following the CLSI 2017 guidelines and using the broth microdilution method. Haemolysis on red blood cells (RBCs) and cytotoxicity on peripheral blood mononuclear cells (PBMCs) were performed to determine the safety of these compounds. The MICs of 3, 4, and 5 against reference strains were 4–64 μg/ml, 2–64 μg/ml, and 0.5–64 μg/ml, respectively. The MICs observed for MRSA were (3) 32 μg/ml, (4) 2–4 μg/ml and (5) 2–4 μg/ml whilst those for VRE were (3) 8–16 μg/ml, (4) 4 μg/ml and (5) 2–16 μg/ml, respectively. In the presence of 50% human serum, there was no significant effect on the MICs. The compounds did not exhibit any effect on cell viability at their effective concentrations. Teixobactin derivatives (3, 4, and 5) inhibited bacterial growth in drug-resistant bacteria and hence emerge as potential antimicrobial agents. Molecular dynamic simulations suggested that the most dominant binding mode of Lys10-teixobactin (4) to lipid II is through the amide protons of the cycle, which is identical to data described in the literature for the natural teixobactin hence predicting the possibility of a similar mechanism of action.

## Introduction

The rate of antibiotic resistance is increasing faster than the development of new compounds for clinical practice. In an extremely short period, resistance to antibiotics has become a significant cause of disease and death globally (Penesyan et al., 2015; Brown and Wright, 2016; Hamilton and Wenlock, 2016). Limited success in collective research efforts to synthesize novel and efficient compounds has contributed to the drug-resistance scenario we are now facing and to the lack of new and efficient treatment options.

The first antibiotics were produced through screening soil microorganisms. However, by the 1960s, this limited resource of cultivable bacteria had been overexploited (Lewis, 2012). Synthetic approaches to produce antibiotics have been unable to replace this platform. Uncultured bacteria, which make up 99% of all species in external environments, emerge as a potent source of new antibiotics (Kaeberlein et al., 2002; Nichols et al., 2010; Fang et al., 2012).

**FIGURE 1:**
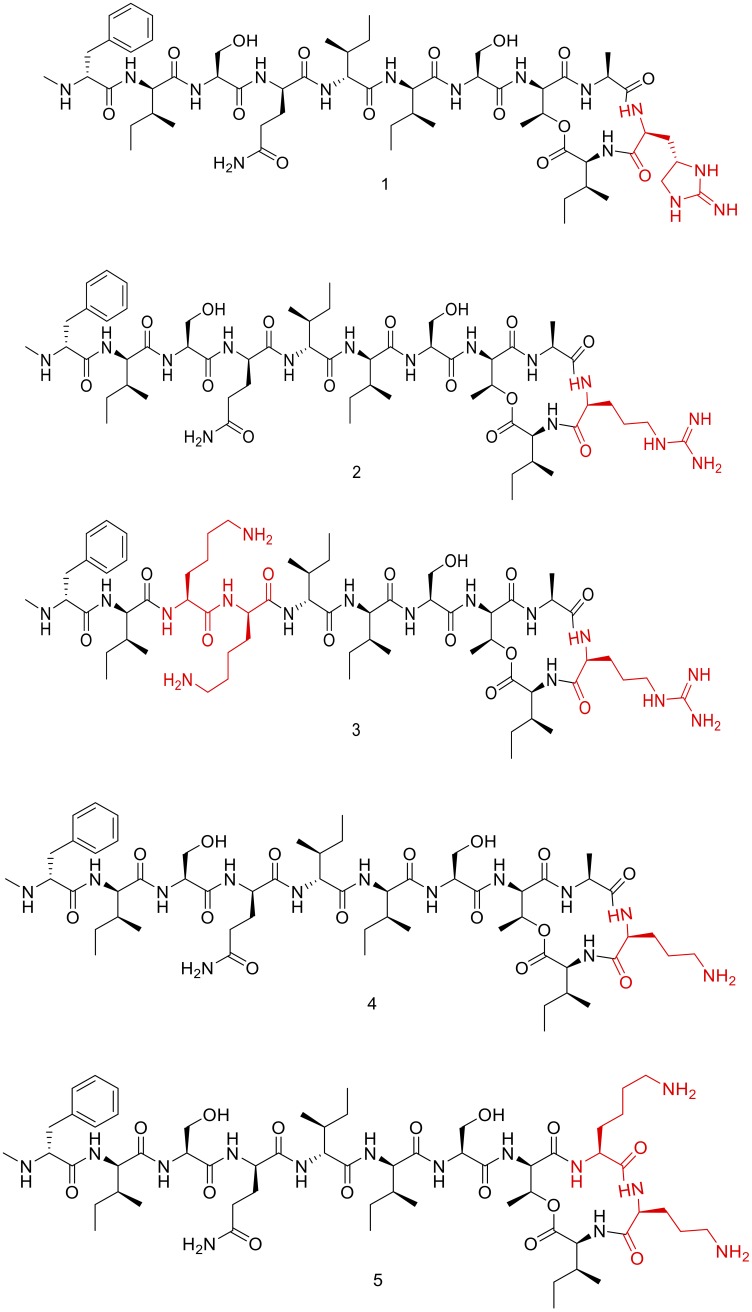
Chemical structure of Teixobactin and derivatives (1, 2, 3, 4, and 5).

Teixobactin (1, **Figure 1**) is a new class of antibiotic that was discovered through the screening of uncultured bacteria using i-Chip (isolation chip), a revolutionary method for bacterial culture (Ling et al., 2015; Piddock, 2015; von Nussbaum and Süssmuth, 2015). Teixobactin was identified as an effective agent against Gram-positive bacteria. It inhibits cell wall synthesis by binding to two lipid cell wall precursors, namely lipid II (peptidoglycan precursor) and lipid III (teichoic acid precursor) (Ling et al., 2015; Homma et al., 2016). Vancomycin also targets lipid II. However, taking into account that Teixobactin has been demonstrated to be active against vancomycin-resistant enterococcus (VRE), its binding is through a different region compared to that of the Vancomycin. In this regard, biochemical assays have demonstrated that teixobactin binds the pyrophosphate and the first sugar moiety present in both, lipid II and lipid III (Ling et al., 2015).

Although much attention has shifted towards combating Gram-negative bacteria, there is still a need for compounds with novel mechanisms and low resistance profiles against Gram-positive strains. In this regard, Texiobactin can satisfy this need and contribute to the treatment of resistant Gram-positive bacteria such as VRE and MRSA. In this study we sought to evaluate three novel derivatives of Teixobactin (3, 4, and 5) and determine their activity against clinically relevant Gram-positive resistant bacteria as well as against Gram-negative species.

## Materials and Methods

### Antibiotics and Reagents

All the derivatives were dissolved in 5% DMSO. GIBCO RPMI-1640 cell culture media (with HEPES, L-glutamine and sodium pyruvate) was obtained from Life Technolgies (Carlsbad, CA, United States). Hyclone fetal bovine serum was purchased from GE Healthcare Life Sciences (Chicago, IL, United States). Phosphate Buffered Saline (PBS) was obtained from Lonza (Basel, Switzerland). Nunclon Delta Surface sterile microtiter plates (including the Edge 2.0 plate) were bought from Thermo Fisher Scientific (Waltham, MA, United States). Human serum from male AB plasma (sterile and filtered), antibiotics, antimycotic solution and all other reagents were obtained from Sigma (St. Louis, MO, United States).

### Bacterial Strains

Clinical isolates of MRSA and VRE were obtained from Lancet Laboratories, Durban, South Africa, with ethical approval BE394/15 from the Biomedical Research Ethical Committee of the University of KwaZulu-Natal. Four reference strains of bacteria, namely *Escherichia coli* ATCC 25922, *Pseudomonas aeruginosa* ATCC 27853, *Bacillus subtilis* ATCC 6051 and *Staphylococcus aureus* ATCC 29213 were obtained from the American Type Culture Collection (ATCC).

### Synthesis, Purification, and Characterization of Teixobactin Derivatives

Our group previously synthesized the Teixobactin derivatives (3, 4, and 5) (**Figure 1**) used in this study. Furthermore, they were chemically characterized by HPLC and MS and subjected to preliminary biological testing against two Gram-positive and two Gram-negative ATCC strains (Abdel Monaim et al., 2017).

### Minimum Inhibition Concentration (MIC) and Minimum Bactericidal Concentration (MBC) Determination

The MICs of the Teixobactin derivatives were determined using the broth microdilution method following the Clinical and Laboratory Standards Institute [CLSI], 2017 guidelines. Two-fold dilutions of each compound solution were prepared using cation adjusted Mueller–Hinton Broth (CAMHB) in a microtiter plate. A 0.5 McFarland-standardized bacterial inoculum was used to prepare a total volume of 200 μl in each microtiter well. The plates were incubated at 37°C for between 18 and 20 h. The MIC was determined as the lowest concentration at which no visible bacterial growth was observed. Control wells for bacteria and media were also included. Meropenem, vancomycin and ampicillin were used as standard control drugs. The plates containing VRE were incubated at 35°C under aerobic conditions. The MBC was determined as the lowest concentration of the test compound that was able to produce a 99.9% decrease in viable bacterial cells on the agar plates. Control wells included the same amount of solvent used in dissolving the drug candidates, medium and bacteria.

### Effect of Human Serum on the MICs

The effect of serum on the MICs was determined in a similar way to the MIC method described above, but in this case 50% human serum: Mueller-Hinton broth was prepared.

### Time-Kill Kinetic Assays

Time-kill assays were performed following CLSI guidelines and previously described methods (Wang et al., 2015; Clinical Laboratory Standard Institute [CLSI], 2017; Zheng et al., 2017). Overnight bacterial cell cultures were suspended in CAMHB and adjusted to an absorbance of approximately 10^6^ CFU/ml. Varying concentrations of the test compounds were added to the inoculum suspensions, with final concentrations corresponding to 1x MIC, 2x MIC, and/or 4x MIC, and incubated at 37°C. Aliquots were removed from the inoculum cultures after 0, 1, 2, 4, 6, 8, and 24 h of incubation. They were then serially diluted, plated on MH agar and incubated for 24 h at 37°C. Bacterial cell viability was determined by colony count. The assays were performed in triplicate. Data was presented as mean and standard deviation of three independent replicates, analyzed with one-way ANOVA followed by Dunnett’s test to determine the significance relative to the untreated bacteria (*P* < 0.05).

### Cell Culture

The Buffy coat used in this study was obtained from the South African National Blood Service (SANBS). The anonymised product is provided by the SANBS for research purposes, upon approval from their Ethics Committee (National Health Laboratory Service Clearance Certificate approval no: 2013/18). Aseptic techniques and appropriate biosafety precautions were observed.

### Haemolysis Assay on Red Blood Cells (RBCs)

The haemolysis assay was performed as previously described (Tramer et al., 2012; Jayamani et al., 2017), with modifications to allow for a 96-well microtiter plate format. Briefly, washed red blood cell pellet was re-suspended in PBS (to obtain a hematocrit of approximately 20%). Next, 10 μL of the cells was aliquoted into a 96-well microtiter plate containing 170 μL of PBS and lysed by addition of 20 μL of 1% Triton^TM^ X-100 solution. After 30 min, the samples were spun at 3000 *g* for 5 min in an Orto Alresa Digicen 21R plate centrifuge. Absorbance was read at 405 nm in a Tecan Sunrise^TM^ plate reader.

Seven serial 5-fold dilutions of the compounds were then prepared in triplicate by adding 25 μL of the compound to 100 μL of PBS. Controls (i.e., 0% and 100% hemolysis samples) were included. Appropriately diluted RBCs (10 μL RBCs and 90 μL PBS per well) were added to the microtiter plate and incubated at 37°C for 30 min and then spun at 3000 *g* for 5 min. The supernatants were then transferred to a fresh microtiter plate, and absorbance was read at 405 nm. The viability of the RBCs at each concentration of the compound was calculated as follows: % viability = 100 × [1 – [A_t_/(A_100_ - A_0_)]] where A_t_ = mean absorbance of the test compound at a given concentration, A_0_ = mean absorbance of the untreated control, and A_100_ = mean absorbance of the sample lysed with Triton^TM^ X-100. The results were represented graphically. The experiment was performed in triplicate (*n* = 3). The error bars indicate the standard deviation. One-way ANOVA followed by Dunnett’s test was performed to determine the significance relative to the untreated RBC (*P* < 0.05; indicated by ^∗^).

### Cytotoxicity on Peripheral Blood Mononuclear Cells (PBMCs)

The cytotoxicity assay was performed as previously described with slight modifications (Pannecouque et al., 2008; Pinto et al., 2011; Araújo et al., 2013; Azumah et al., 2016). Briefly, PBMCs (100,000 viable cells/well) were placed into the wells of a Nunclon^TM^ Delta Surface Edge 2.0 microtiter plate containing 100 μl of RPMI-1640 with 10% fetal bovine serum, 1% Antibiotic Antimycotic solution and 3% phytohemagglutinin. The cells were then incubated for 24 h at 37°C and 5% CO_2_. Seven serial 5-fold dilutions of test compounds were prepared and transferred to the appropriate wells of the plate containing the cells. The plate was then incubated for 72 h at 37°C and 5% CO_2_. Thereafter, 20 μl of MTT salt (7.5 mg/ml) was added to each well, and the plate was incubated for a further 4 h. Then 100 μl of the media was carefully removed from each well (avoiding agitation of the crystals) and replaced with 100 μl of solubilisation solution (containing acidified isopropanol and Triton^TM^ X-100). The plate was then placed on a shaker for 30 min to facilitate dissolution of the crystals. Absorbance was read at 550 nm (background: 690 nm). The results were shown graphically. The experiment was performed in triplicate (*n* = 3). The error bars indicate the standard deviation. One-way ANOVA followed by Dunnett’s test was performed to determine the significance relative to the untreated PMBC (*P* < 0.05; indicated by ^∗^).

### Molecular Dynamics Simulation

Structures of lipid II and teixobactin were downloaded from Automated Topology Builder (ATB) and Repository (Malde et al., 2011). In the teixobactin structure, the residue of enduracididine was replaced by Lys by molefacture program of VMD to get Lys_10_-teixobactin (4) structure (Humphrey et al., 1996). CHARMM General Force Field (CGenFF) parameters were used for simulation of both molecules (Vanommeslaeghe et al., 2010). The molecular dynamics simulation system contains one lipid II molecule, one Lys10-teixobactin (4) molecule, and 6767 water molecules. The TIP3P water model was used for the water molecules (Mark and Nilsson, 2001). The system was first energy minimized using the steepest descent algorithm (Bixon and Lifson, 1967), after which two sequential equilibrations were performed using canonical ensemble (NVT), followed by an isobaric-isothermic ensemble (NPT) for 100 picoseconds (ps) each, and production simulation was performed using NPT ensemble for 100 nanoseconds (ns). The simulation was performed at 310 K temperature and 1 atm pressure, for temperature coupling velocity-rescale method and for pressure coupling the Parrinello-Rahman method was used (Parrinello and Rahman, 1981). The Particle Mesh Ewald method was used for long-range electrostatic interactions (Darden et al., 1993), with 10Å cut-off being used to calculate the VdW and short-range coulombic interactions. MD simulation was performed using the GROMACS simulation package (Abraham et al., 2015). To identify the binding region of lipid II with Lys_10_-teixobactin (4) an *in-house* TCL script was used. Script counts the numbers of frames that each oxygens atoms of lipid II were within 3.5 Å of protons of Lys10-teixobactin (4) throughout the simulation time.

## Results

### Teixobactin Derivatives

Teixobactin is an 11-amino acid “head to side-chain” cyclodepsipeptide (**Figure 1**) with a D-Thr as a bridge head that forms the ester with the carboxylic group of a L-Ile. L-Ala and the post-translational modified L-*allo-*enduracidine (End), which contains a cyclic guanidine, are also part of the cycle (Abdel Monaim et al., 2016a; Dhara et al., 2016; Giltrap et al., 2016; Jin et al., 2016, 2017; Yang et al., 2016, 2017; Monaim et al., 2017; Parmar et al., 2017a,b; Schumacher et al., 2017; Wu et al., 2017). The tail is formed by two moieties of L-Ser, two moieties of L-Ile, D-*allo-*Ile and D-Gln ending with a *N-*Me-D-Phe. As L-*allo-*End was not commercially available, our group concentrated their efforts on synthesizing Arg_10_-Teixobactin (2, **Figure 1**), in which the L-*allo-*End is substituted by Arg (Jad et al., 2015; Parmar et al., 2016).

Arg_10_-Teixobactin (2), which has been converted from the parent Teixobactin analog, has slightly lower activity than Teixobactin. Our group had previously used a Lys-scanning strategy to prepare a small library of Teixobactin analogs containing more than one Lys residue—a residue that is absent in the natural structure (Abdel Monaim et al., 2016b, 2017). From this collection of peptides, three (3, 4, and 5, **Figure 1**) with good MICs against sensitive bacteria (ATCC strains) were selected for further *in vitro* evaluation in this study.

### Antimicrobial Activity of Teixobactin Derivatives and the Effects of Human Serum on the MICs

The antimicrobial activity of the three derivatives (3, 4, and 5) was examined by *in vitro* screening against drug-resistant and -sensitive bacteria using the broth micro-dilution method, following CSLI guidelines. The derivatives inhibited sensitive Gram-positive (ATCC strains) and resistant MRSA and VRE isolates (**Tables 1–3**). They demonstrated potent antimicrobial activity against Gram-positive bacteria as opposed to Gram-negative bacteria. Three conventional antibiotics (meropenem, vancomycin and ampicillin) were used as controls and exhibited activity against the drug-sensitive ATCC strains. The following MIC_50_ were recorded for the derivatives: (3) 32 μg/ml, (4/5) 2 μg/ml for MRSA, as well as (3) 16 μg/ml and (4/5) 4 μg/ml for VRE. The derivatives yielded MICs as low as 2 μg/ml and 0.5 μg/ml for Gram-positive reference ATCC strains *S. aureus* and *B. subtilis* respectively. The MICs of the experimental compounds against susceptible Gram-negative bacteria were 32 μg/ml for *E. coli* and 64 μg/ml for *P. aeruginosa*. These compounds also inhibited drug-resistant clinical isolates of MRSA at concentrations of 32 μg/ml, 4 μg/ml and 2 μg/ml for 3, 5, and 4 respectively. Vancomycin, the current drug of choice for the treatment of MRSA, had an MIC of 1 μg/ml; while the MIC of ampicillin against MRSA was ≥512 μg/ml. Compounds 3, 4, and 5 showed MICs against VRE of 16 μg/ml, 8 μg/ml, and 4 μg/ml and respectively.

**Table 1:** Minimum inhibitory concentration (MIC), minimum bactericidal concentration (MBC) and MIC in presence of 50% human serum of Teixobactin derivatives against susceptible reference strains of bacteria.

Antimicrobial agents	Organism											
	Gram-positive						Gram-negative					
	*S. aureus ATCC 29213*			*B. subtilis ATCC 6051*			*E. coli ATCC 25922*			*P. aeruginosa ATCC 27853*		
	MIC (μg/ml)	MBC (μg/ml)	50% serum (MIC μg/ml)	MIC (μg/ml)	MBC (μg/ml)	50% serum (MIC μg/ml)	MIC (μg/ml)	MBC (μg/ml)	50% serum (MIC μg/ml)	MIC (μg/ml)	MBC (μg/ml)	50% serum (MIC μg/ml)
3	32	64	64	4	8	2	64	64	>64	64	128	>64
4	4	16	4	2	8	1	64	64	>64	>64	128	>64
5	2	8	4	0.5	1	0.5	32	64	>64	64	128	>64
Meropenem	0.25	ND	ND	0.125	ND	ND	0.125	ND	ND	1	ND	ND

**Table 2:** Minimum inhibitory concentration of Teixobactin derivatives against methicillin-resistant *Staphylococcus aureus* (MRSA).

Isolates	Origin^a^	Species	3	4	5	Vancomycin	Ampicillin
			
			MIC (μg/ml)	MIC (μg/ml)	MIC (μg/ml)	MIC (μg/ml)	MIC (μg/ml)
B11970	Blood	*S. aureus*	32	2	2	1	>512
P10781	Nasal	*S. aureus*	32	2	2	1	>512
P10747	CVP	*S. aureus*	32	2	2	1	>512
S37938	–	*S. aureus*	32	2	2	1	>512
S18155	ETT	*S. aureus*	32	2	2	0.5	>512
B13178	Blood	*S. aureus*	32	2	2	1	>512
440260	–	*S. aureus*	32	4	4	1	>512
S18970	–	*S. aureus*	32	2	2	1	>512
P11520	Pus	*S. aureus*	32	4	4	1	512
T5683	Nasal	*S. aureus*	32	2	2	1	>512
MIC50			32	2	2	1	>512

*^a^ETT, Endotracheal tube; CVP, Central venous catheter, –, Missing data.*

**Table 3:** Minimum inhibitory concentration of Teixobactin derivatives against vancomycin-resistant enterococci (VRE).

Isolates	Species	3	4	5	Vancomycin
		
		MIC (μg/ml)	MIC (μg/ml)	MIC (μg/ml)	MIC (μg/ml)
951245262 (A)	*Enterococcus faecium*	8	4	4	>128
951234856 (B)	*Enterococcus faecium*	16	4	4	>128
951208931 (C)	*Enterococcus faecium*	16	4	4	>128
938636470 (D)	*Enterococcus faecium*	16	8	4	>128
938666613 (E)	*Enterococcus faecium*	16	16	4	>128
938600912 (F)	*Enterococcus faecium*	16	2	8	>128
938072607 (G)	*Enterococcus faecium*	16	8	4	>128
944414000 (H)	*Enterococcus faecium*	16	8	4	>128
945530665 (I)	*Enterococcus faecium*	16	4	4	>128
U43821 (J)	*Enterococcus faecium*	16	8	4	>128
*MIC50*		16	4	4	>128

No significant effect of serum on the MICs was observed when the reference bacterial strains were tested with varying concentrations of the derivatives in the presence of 50% human serum; the values varied by only ±1 in fold dilutions. Compounds 3, 4, and 5 demonstrated bactericidal activities, yielding a 99.9% decrease in viable cells on the agar plates at concentrations ≤ 4x MIC values. All the experiments were conducted in triplicate to confirm the outcomes.

### Time-Kill Kinetics

**FIGURE 2:**
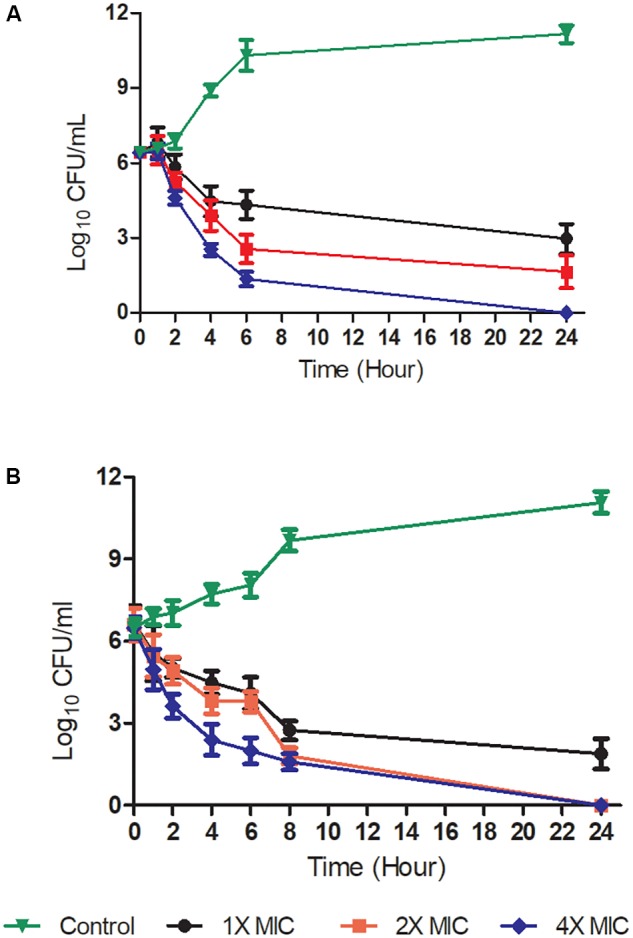
Time-kill kinetics at a range of concentrations of Teixobactin derivative 4. Gram-positive bacteria [*S. aureus* ATCC 29213 **(A)** and *B. subtilis* ATCC 6051 **(B)**] were challenged with compound 4 at 1X, 2X, and 4X MIC levels. The experiment was performed in triplicate (*n* = 3). Data was presented as mean and standard deviation of three independent replicates, analyzed with one-way ANOVA followed by Dunnett’s test to determine the significance relative to the untreated bacteria (*P* < 0.05).

Time-kill kinetic assays were performed to determine whether the Teixobactin derivatives showed time-dependent or concentration-dependent properties, as well as whether their effects were bacteriostatic or bactericidal. The time-kill curves of compound 4 against Gram-positive *S. aureus* ATCC 29213 and *B. subtilis* ATCC 6051 are shown in **Figure 2**. The kinetics indicated time- and concentration- dependent bacterial killing for this compound, and the bactericidal effect was observed at a concentration of 2x and 4x MIC levels at 6 h, as well as at 1x MIC at 24 h against *S. aureus* and *B. subtilis*. Exposure of *S. aureus* and *B. subtilis* to 4 at 2x and 4x MIC resulted in a decrease in bacterial cell count greater than 3 log_10_ relative to the initial density from 6 and 4 h respectively, which was also indicative of a bactericidal effect. At a concentration of 1x MIC, compound 4 caused a significant reduction in log_10_ CFU 6 h after its addition (**Figure 2**).

### Haemolysis and Cytotoxicity

**FIGURE 3:**
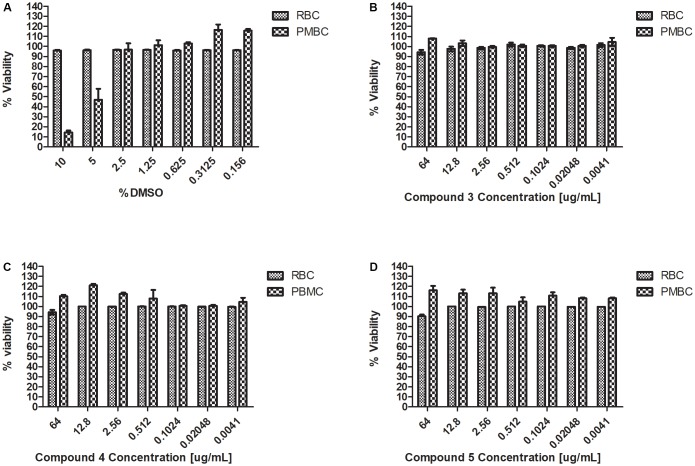
Haemolysis and cytotoxicity effects of Teixobactin derivatives at various concentrations **(A)** Viability (%) of RBCs and PBMCs treated with DMSO; **(B)** Viability (%) of RBCs and PBMCs treated with compound 3; **(C)** Viability (%) of RBCs and PBMCs treated with compound 4; **(D)** Viability (%) of RBCs and PBMCs treated with compound 5. The experiment was performed in triplicate (*n* = 3). The error bars indicate the standard deviation. One-way ANOVA followed by Dunnett’s test was performed to determine the significance relative to the RBC and PMBC (*P* < 0.05).

Haemolysis and cytotoxicity effects were evaluated by exposing RBCs and PBMCs to varying concentrations of the Teixobactin derivatives. The concentrations tested showed no cytotoxic effect on PBMCs or any hemolytic effect on erythrocytes. RBC and PBMC viability was above 90% at the highest concentration of the derivatives used in this study (64 μg/mL) (**Figure 3**).

### Molecular Dynamics Simulation: Binding of Lys10-Teixobactin (4) With Lipid II

**FIGURE 4:**
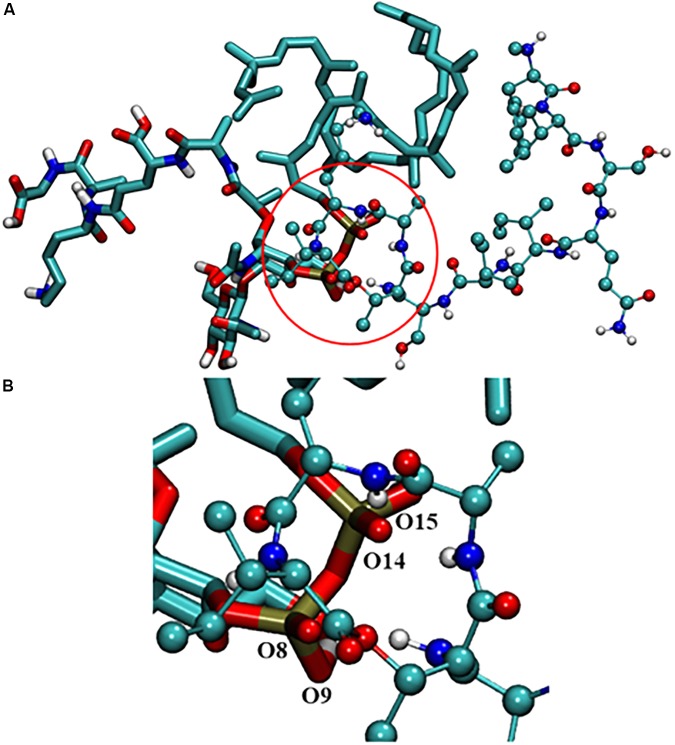
**(A)** Interaction between lipid II and Lys10-teixobactin (4); **(B)** magnification of the pyrophosphate and the cycle. For clarity purposes, only polar hydrogens were shown.

To understand the binding modes of Lys_10_-teixobactin (4) with lipid II, 100 nanoseconds (ns) molecular dynamics simulations were performed. The data revealed different binding modes of Lipid II with Lys10-teixobactin (4) out of which pyrophosphate interaction with amide group proton of the cycle was the most dominant. To identify the interacting region of lipid II with Lys10-teixobactin (4), the number of frames that oxygens of lipid II is within 3.5 Å (hydrogen bonding distance) of proton atoms of Lys10-teixobactin (4) for whole simulations was calculated (**Table 4** indicates > 1000). As there are 10000 frames (10ps each), it was observed that four oxygens of pyrophosphate groups were formed the most interaction with Lys10-teixobactin (4) cycle amide group protons (**Figure 4**).

**Table 4:** Number of frames (>1000) that lipid I oxygens was within 3.5 Å of Lys10-teixobactin (4) protons.

Oxygens of lipid II (Atom number)	Number of frames
Pyrophosphate oxygen (O14)^a^	3518
Pyrophosphate oxygen (O15)	2772
Pyrophosphate oxygen (O8)	2676
Pyrophosphate oxygen (O9)	1713
Oxygen acetyl of sugar moiety 1	1447
Ala-6 oxygen carbonyl group	1066
Glu-7 oxygen α-carboxyl group	1050

*^a^The number of the O atoms are shown in the **Figure 4B**.*

## Discussion

Although antibiotic resistance in Gram-positive bacteria is increasing worldwide, as indicated by the WHO list of high-priority pathogens (i.e., VRE and MRSA), much attention has shifted to combating Gram-negative bacteria. Teixobactin has been demonstrated as effective against Gram-positive bacteria and no detectable resistance has been reported yet. The capacity of Teixobactin is attributed to it being structurally distinct from glycopeptides and it being the first member of a new class of lipid II binding antibiotics.

The MICs of compounds 4 and 5 for reference strains *S. aureus* and *B. subtilis* were between 0.5 and 4 μg/ml (**Table 1**) while for the MRSA isolates they were between 2 and 4 μg/ml. Compound 3 had an MIC of 32 μg/ml, a value that was much higher than that reported for the control antibiotic vancomycin (0.5–1 μg/ml). Other groups reported compound 3 to have a MIC of 4 μg/ml against MRSA (Jin et al., 2016; Parmar et al., 2017a; Schumacher et al., 2017). These results were echoed in the present study, as the MIC observed against MRSA for this compound was between 2 and 4 μg/ml.

The MBC reported by Ling et al. (2015) was 2x the MIC of Teixobactin. The bactericidal activity of Teixobactin and its derivatives against Gram-positive bacteria is superior to that of vancomycin, and these compounds retain excellent bactericidal activity against VRE (Ling et al., 2015). The strong bactericidal activity of Teixobactin and its derivatives is attributed to not only inhibition of peptidoglycan synthesis but also the synergistic inhibition of cell wall teichoic acid synthesis. These derivatives show the same bactericidal activity as that observed for Teixobactin and the MIC/MBC ratios were ≤4 for all the three derivatives.

Time-kill kinetic assays were carried out with compound 4 as it showed the best MICs against *S. aureus* and *B. subtilis*. Complete bactericidal activity was observed at concentrations of 16 and 8 μg/ml at 4 h. Similar to observations of other Teixobactin derivatives, compounds 3, 4, and 5 had no cytotoxic or hemolytic effect *in vitro.* In the presence of 50% serum, there was no drastic change in the MICs (**Table 1**).

On the basis of our observations, we can conclude that human serum has no effect on the antibacterial activity of compounds 3, 4, and 5. These results are similar to those observed by Parmar et al. (2017a). The serum effect is essential as it aids in speculating the probable *in vivo* activity of the drug. These derivatives will possibly have low protein binding properties because they bind to multiple target sites, none of which are proteins. The present study confirms that Teixobactin derivatives 3, 4, and 5 are safe and can thus be considered potential treatment options against resistant bacterial infections (VRE and MRSA).

Interestingly, we observed that, at higher concentrations, 3, 4, and 5 were also active against Gram-negative bacteria (**Table 1**). This is a relevant observation given the low toxicity of these compounds. These derivatives may exert their activity against Gram-negative bacteria by disrupting the outer membrane layer. In conclusion, we have demonstrated the highly potent antimicrobial activity of three Teixobactin derivatives against clinically significant isolates of bacteria. Unlike vancomycin, these derivatives showed early stage killing kinetics.

Due to the lack of crystallography or NMR data of the complex lipid II-teixobactin, until now it has not been possible to establish the interaction of teixobactin residues with lipid II experimentally. However, Lewis and co-workers (Ling et al., 2015) have hypothesized that lipid II pyrophosphate group and *N*-acetylmuramic acid are essential for the binding to teixobactin. This was further supported by recent MD simulation study of Liu et al. (2017) that showed the importance of the participation of the oxygens of pyrophosphate group of lipid II with amide protons of the teixobactin cycle. In the MD simulation study carried out herein, similar interactions were also observed with Lys10-teixobactin (4) and pyrophosphate of lipid II (**Table 4** and **Figure 4**). These data suggest that the most dominant binding mode of Lys10-teixobactin (4) to lipid II is through the amide protons of the cycle, which is identical to data described in the literature for the natural teixobactin hence predicting the possibility of a similar mechanism of action.

Given these promising results, further research should address the mechanism/s of action exerted by these compounds. The findings of this study will contribute to the development of other Teixobactin derivatives with high potent antimicrobial activity against resistant bacterial strains and to the development of novel peptide-based antimicrobial agents to tackle the global threat of drug resistance.

## Author Contributions

ER, AS, SM, DA, BT, and FA conceived and designed the experiments. ER, AS, SM, DA, HK, and NA performed the experiments. ER, AS, DA, RP, HK, and NA analyzed the data. RP, BT, FA, and LB contributed to reagents, materials, and analysis tools. ER wrote the paper. All authors did a critical revision of the manuscript.

## Conflict of Interest Statement

The authors declare that the research was conducted in the absence of any commercial or financial relationships that could be construed as a potential conflict of interest.
